# Variability of seizure-like activity in an *in vitro* model of epilepsy depends on the electrical recording method

**DOI:** 10.1016/j.heliyon.2020.e05587

**Published:** 2020-11-27

**Authors:** Shabnam Ghiasvand, Chris R. Dussourd, Jing Liu, Yu Song, Yevgeny Berdichevsky

**Affiliations:** a Bioengineering Lehigh University, United States; b Electrical Engineering Lehigh University, United States

**Keywords:** Epilepsy, Seizure similarity, Local field potential, Microwire, MEA, Jitter, Organotypic hippocampal cultures, Neuroscience, Tissue culture, Pathophysiology, Physiology, Neurology

## Abstract

**Background:**

Hippocampal and cortical slice-based models are widely used to study seizures and epilepsy. Seizure detection and quantification are essential components for studying mechanisms of epilepsy and assessing therapeutic interventions. To obtain meaningful signals and maximize experimental throughput, variability should be minimized. Some electrical recording methods require insertion of an electrode into neuronal tissue, change in slice chemical microenvironment, and transients in temperature and pH. These perturbations can cause acute and long-term alterations of the neuronal network which may be reflected in the variability of the recorded signal.

**New method:**

In this study we investigated the effect of experimental perturbations in three local field potential (LFP) recording methods including substrate micro-wires (s-MWs), multiple electrode arrays (MEAs), and inserted micro wire electrodes (i-MW). These methods enabled us to isolate effects of different perturbations. We used organotypic hippocampal slices (OHCs) as an in-vitro model of posttraumatic epilepsy. To investigate the effect of the disturbances caused by the recording method on the paroxysmal events, we introduced jitter analysis, which is sensitive to small differences in the seizure spike timing.

**Results:**

Medium replacement can introduce long-lasting perturbations. Electrode insertion increased variability on a shorter time scale. OHCs also underwent spontaneous state transitions characterized by transient increases in variability.

**Comparison with existing methods:**

This new method of seizure waveform analysis allows for more sensitive assessment of variability of ictal events than simply measuring seizure frequency and duration.

**Conclusion:**

We demonstrated that some of the variability in OHC recordings are due to experimental perturbations while some are spontaneous and independent of recording method.

## 1 Introduction

Hippocampal and cortical slice-based models are widely used to study seizures and epilepsy (Pitkänen et al., 2017). In acute models, seizures are provoked by 4-aminopyrodine (4-AP), high extracellular concentration of K^+^, low extracellular concentrations of Mg^2+^, and other methods (Heuzeroth et al., 2019; Campos et al., 2018; Nakagawa and Durand, 1991). In a chronic model, slices are maintained *in vitro* to allow epileptogenic processes, induced by slicing injury, to run their course, and seizures appear spontaneously (Lillis et al., 2015; Dyhrfjeld-Johnsen et al., 2010). Slice-based models are used not only for understanding basic mechanisms of epilepsy, but also to discover new drugs and treatments for this debilitating disorder. One important constraint that determines a model's usefulness is its experimental throughput. This, in part, depends on the length and the number of the experiments that are required to achieve statistically meaningful results. We and others have developed platforms that enable multiple slice model-based experiments to be run in parallel (Liu et al., 2016; Stopps et al., 2004; Kroker, et al., 2011; Graef et al., 2013). However, experimental variability may be a limiting factor in the maximum throughput that can be achieved with these technological solutions. If variability is high relative to the size of the effect that is being studied, large numbers of experiments may be required to achieve statistical power, and effective throughput of even highly parallel platforms may be limited.

In our previous studies using the chronic hippocampal slice culture model of epilepsy, we noticed that experimental variability may be a function of the assay used to assess seizure load, rather than of the model itself. For example, measurements of lactate accumulation in culture medium typically had a smaller variance to mean ratio compared to direct electrical assessment of seizures with a microelectrode (Berdichevsky et al., 2013). Since lactate measurements are only a biomarker of seizure load (During et al., 1994), electrical measurements were required to confirm biomarker results. This resulted in a significant throughput bottleneck for drug discovery screens (Berdichevsky et al., 2016). We then noticed that the variance of electrical measurements of seizures heavily depends on the electrical recording method. The lower variance to mean (Fano factor) of seizure frequency and duration for measurements with a multiple electrode array (MEA) enabled use of fewer samples compared to measurements with an inserted microelectrode (Liu et al., 2019a).

Electrical recordings typically require placement of slices into recording solution maintained at a certain temperature and pH and the positioning of a microelectrode into close contact with neurons within the slice. Slices thus undergo temperature and pH transients (Hedrick and Waters, 2011; Hedrick and Waters, 2012; Church, 1999), significant changes in the composition of their microenvironment (ion, glucose, amino acid concentrations (Liu et al., 2017)), and suffer electrode insertion damage prior to a recording session. A widely accepted procedure is to allow slices to ‘stabilize’ prior to recording data; however, it is possible that some stabilization time constants may be comparable to or longer than typical recording time. For example, electrode insertion may cause damage to both neurons and their processes, and cause tissue response that may involve inflammation and glial cell activation on time scale of days (Potter et al., 2012; Kozai et al., 2015; Ravikumar et al., 2014; McConnell et al., 2009; Wellman and Kozai, 2017; Biran et al., 2005; Eles et al., 2018). Artificial cerebrospinal fluid that is typically used for recording does not contain many of the amino acids, proteins, and other biomolecules present in the cerebrospinal fluid (Liu et al., 2017). Transition of slices into this artificial environment may shift cellular metabolism of both neurons and astrocytes, and result in long term changes to neural circuit function (Patel, 2018). Even in long term slice cultures, transition of slices into fresh culture medium prior to a recording session may transiently remove growth factors and other biomolecules that cells have secreted into the medium.

Changes to the slice environment may result in increased variability of seizure duration and frequency owing to effects on seizure initiation, dynamics, and termination. Therefore it is important to investigate which aspects of experimental methods significantly contribute to variability, in order to determine the method of electrical recording that will have the least variability. In this work, we quantified spontaneous electrogenic seizures in organotypic hippocampal cultures (OHCs) in order to assess variability under different recording conditions. OHCs have been recognized as an *in-vitro* model of posttraumatic epilepsy. These cultures become spontaneously epileptic after being cultured for approximately 7 days *in-vitro* (DIV) (Berdichevsky et al., 2017; Lillis et al., 2015; Dyhrfjeld-Johnsen et al., 2010). We used three methods of local field potential recordings to detect seizures. In the first method, slices were removed from culture dishes on the recording day, placed into a recording chamber, and had a single tungsten microwire electrode inserted into the pyramidal layer for seizure detection. We termed this method “inserted microwire”, or i-MW. In the second method, slices were cultured on microwires glued to the bottom of the culture dishes (Liu et al., 2019b). This method avoids the need to damage slices for recording by microwire insertion, and slices can be recorded in the culture dish without exchanging medium. We termed this method “substrate-integrated microwire”, or s-MW. To expand the recording time period and continuously monitor the seizures, a third method was used. We cultured organotypic slices on multiple electrode arrays (MEAs).

We developed a new seizure waveform analysis method in this work to enable sensitive assessment of seizure variability. The period between each successive paroxysmal spike within a seizure was measured. Each seizure was then characterized by a sequence of inter-spike intervals. We then determined the average difference in spike timing (jitter) between different seizures, or during different recording periods containing multiple seizures (described in more detail in the Methods section). Lower jitter signified low variability between seizures. Jitter analysis is sensitive to small changes in seizure spike timing sequences, and proved to be a robust method for tracking time-dependent changes in seizure variability. We compared jitter in recordings obtained with three different recording methods, and identified the most significant experimental sources of variability.

## 2 Materials and methods

### 2.1 Organotypic hippocampal cultures

Slices of 350 μm thickness were made using McIlwain tissue chopper (Mickle Laboratory Eng. Co., Surrey, United Kingdom), from the hippocampi of Sprague-Dawley rats of post-natal days 7–8. Tissue cultures were then transferred to a poly-D-lysine (PDL) coated substrates. For substrate microwire recordings (s-MW), 6-well plates (Falcon) were used. MEA recordings were collected from micro-electrode arrays (MEA) (60MEA 200/30 IR-TI, Multichannel systems). For optical recordings, 35mm petri-dishes (Falcon) were used as substrates. In the case of single inserted electrode recordings (i-MW), slices were placed on glass coverslips pre-coated with PDL and cultured in 6-well plates. Slices were maintained in a humidified 37 °C incubator with 5% CO_2_ on a rocking platform. A serum free culture medium consisted of Neurobasal-A/B27, 30 μg/ml gentamicin, and 0.5 mM GlutaMAX (Invitrogen). Culture media was changed twice per week. All animal use protocols were approved by the Institution Animal Care and Use Committee (IACUC) at Lehigh University and were conducted in accordance with the United States Public Health Service Policy on Humane Care and Use of Laboratory Animals.

### 2.2 Substrate-integrated microwire recording (s-MW)

For each well of a 6-well plate two PFA-coated tungsten wires (bare diameter = 50.8 μm, coated diameter = 101.6 μm, A-M systems Inc.) were cut at about a length of 15 cm and sterilized with 70% ethanol. The tip of the reference electrode was flamed for about 1cm and was placed at the edge of the well while resting on the bottom of the dish. The recording electrode was positioned in the center of the well with a silicone adhesive (4300 RTV, Bluestar Silicones). Adhesive was cured at 65 °C overnight. Afterwards, the plates were coated with poly-d-Lysine (PDL, Sigma) in a humidified atmosphere at 37 °C overnight. The PDL was then washed away with sterile water. The wells were then filled with NeurobasalA/B27 medium (Thermo Fisher Scientific) and incubated for at least 3 h before dissection. Organotypic slices were then centered on the recording electrode such that the tip was underneath CA3 or CA1 neuronal layers and maintained for two weeks or more.

LFP recordings were acquired by transferring the plates to a recording chamber (Bioscience Tools) connected to a controller to maintain conditions of 37 °C, 5% CO_2_, 21% oxygen, and balanced Nitrogen (Airgas). Extracellular field potentials were collected over 45 min with a high-impedance multiple-channel pre-amplifier stage (PZ2-64, Tucker Davis Technologies) connected to a RZ2 amplifier (Tucker Davis Technologies). Signals were filtered with a band-pass filter (1Hz-3KHz, gain x1000) and sampled at 6 kHz. Recordings were then analyzed using OpenX software (Tucker Davis Technologies) and MATLAB (MathWorks).

### 2.3 MEA devices recording

For the chronic LFP recordings from OHCs, we cultured slices on MEA devices. The electrode contact pads of MEA device were connected to a 16-channel extracellular amplifier with a high impedance head stage (3600, A-M Systems). Signals were digitized with a multiple-channel digital acquisition board (Measurement Computing). The sampling rate was 5 kHz. Data was collected with dClamp software and analyzed using Matlab (Mathworks). Electrical noise was removed by applying a 4th order Butterworth band-stop filter at a frequency of 60Hz. And an additional 4th order Butterworth was applied to remove DC components and mechanical disturbances at frequencies below the 3Hz range.

### 2.4 Inserted microwire recordings (i-MW)

Single electrode recordings were collected from hippocampal slices after seven to fifteen DIV. Slices designated for i-MW were cultured on 24 × 30mm glass coverslips (Electron Microscopy Sciences) allowing slices to be transferred easily to the interface recording chamber. On the day of recording, the coverslip was transferred to a Petri dish, covered partially by artificial cerebrospinal fluid (ACSF, 95% O_2_ and 5% CO_2_) and transferred to the interface chamber. ACSF contained 126 mM NaCl, 25 mM NaHCO_3_, 3.5 mM KCl, 1.3 mM MgCl_2_, 2 mM CaCl_2_, and 11 mM D-glucose. The temperature of the chamber was kept at 37 ° C using a bath circulator, and air was bubbled through the chamber to maintain the pH of the culture medium. Tungsten 0.1MΩ microelectrode was inserted into the CA1 or CA3 region of the hippocampus using a micromanipulator. Recordings under these conditions lasted one hour. Recordings were amplified by 1000x, band-pass filtered 1 Hz to 5kHz and digitized using multichannel analog-to-digital acquisition board and LabView software (National Instruments).

### 2.5 Seizure and spike detection algorithm

Seizures were defined as paroxysmal events (with significantly larger amplitudes than background activity) that occurred for at least 10 s with an event frequency of at least 2 Hz. Color raster plots were generated by binning the data every 0.5 s and calculating the number of super-threshold bins (represent ictal events) per 10 s sliding window. The threshold was set at 20~65 μV above the background non-paroxysmal activity.

Spikes that make up the seizure waveform were detected by considering a lower and upper threshold. These two values were adjustable depending on the recording status but generally the upper threshold was set to 1 mV and lower threshold to 0.5 mV. Any activity that started below 0.5 mV, reached greater than 1 mV, and then dipped down below 0.5 mV in a time period between 5 ms and 150 ms was a potential population spike. The number of spikes detected was narrowed down further by making sure the inter-spike interval was greater than 100 ms. If neural activity reached greater than 1 mV twice within a 100 ms time period, this was likely the result of biphasic spike (White et al., 2006). We recorded the biphasic spikes as one spike using the larger peak as the spike time. Any neural activity that met these criteria was considered a potential spike, and the peak value of activity was recorded as a spike time. The spike detection results from this automatic algorithm (implemented in Matlab) were further confirmed through a graphical user interface where the missing spikes or mistakenly chosen spikes were manually corrected.

### 2.6 Jitter analysis

In order to quantify the small changes in seizure waveforms, we defined the term jitter which effectively measures the difference in seizure spike times. Seizures were aligned to one another according/to a chosen spike time from each of the events. The alignment spike was determined automatically in such a way that would result in the minimum jitter between seizures. The jitter for all spikes in the seizures could then be computed by taking the difference of the inter-spike interval divided by the average of the inter-spike interval. In the equation below, n_interval_ equals the number of intervals in a seizure (total number of spikes-1). If the number of intervals is different between the two seizures, then it equals the number intervals of the seizure with the least number of spikes.

(1) $$Jitter_{seizure1,seizure2}=\frac{\sum_{i=1}^{n_{interval}}\frac{abs\left(SpikeInterval_{seizure1,i}-SpikeInterval_{seizure2,i}\right)}{\left(SpikeInterval_{seizure1,i}+SpikeInterval_{seizure2,i}\right)/2}}{n_{interval}}$$

Effect of downsampling on jitter quantification was examined. Recordings for s-MW with the highest sampling rate of 6 kHz were down-sampled to 1 kHz. Jitter values were recalculated for these recordings. Downsampling did not significantly affect jitter values (effect was less than 10% of the calculated jitter value).

### 2.7 Optical Ca^2+^ recording

jRGECO1a is a genetically encoded calcium indicator was used to trace calcium rise upon insertion of the tungsten electrode (Dana et al., 2016). pAAV.Syn.NES-jRGECO1a.WPRE.SV40 was a gift from Douglas Kim & GENIE Project (Addgene plasmid # 100854; http://n2t.net/addgene:100854; RRID:Addgene_100854) and was applied to culture media on 0 DIV after 1 h incubation post dissection at final concentration of about 10^10^ genome copies/mL. Half of the culture media was replaced with fresh media after 3 DIV and exchanged completely with fresh media on the following media exchange days.

At the time of recording, culture media was replaced with recording medium, which consisted of 138.8 mM NaCl, 2.4 mM KCl, 10 mM HEPES, 10 mM Glucose, 1 mM MgCl_2_, 2 mM CaCl_2_, and 1.2 mM Na_3_PO_4_. 35mm petri-dishes were transferred to the recording stage where the temperature was controlled at 37 °C. Recordings were taken with 10X objective by camera at 5 fps for one hour. Recorded videos were analyzed initially in ImageJ and two regions of interest (ROI) were selected using ROI manager plugin. For each recording, one ROI was chosen at the site where electrode was inserted and one away from the affected region. ROI size was chosen such that the whole area with prolonged elevated calcium was covered. Mean gray values for each ROI were calculated through the entire video and were analyzed further in MATLAB. Asymmetric least square smoothing method was used to determine the baseline (F0) (Eilers and Boelens, 2005). Signal to baseline ratio was calculated as (F(t)-F0)/F0, where F(t) is the raw signal.

## 3 Results

### 3.1 Recording of seizures with three recording platforms

**Figure 1:**
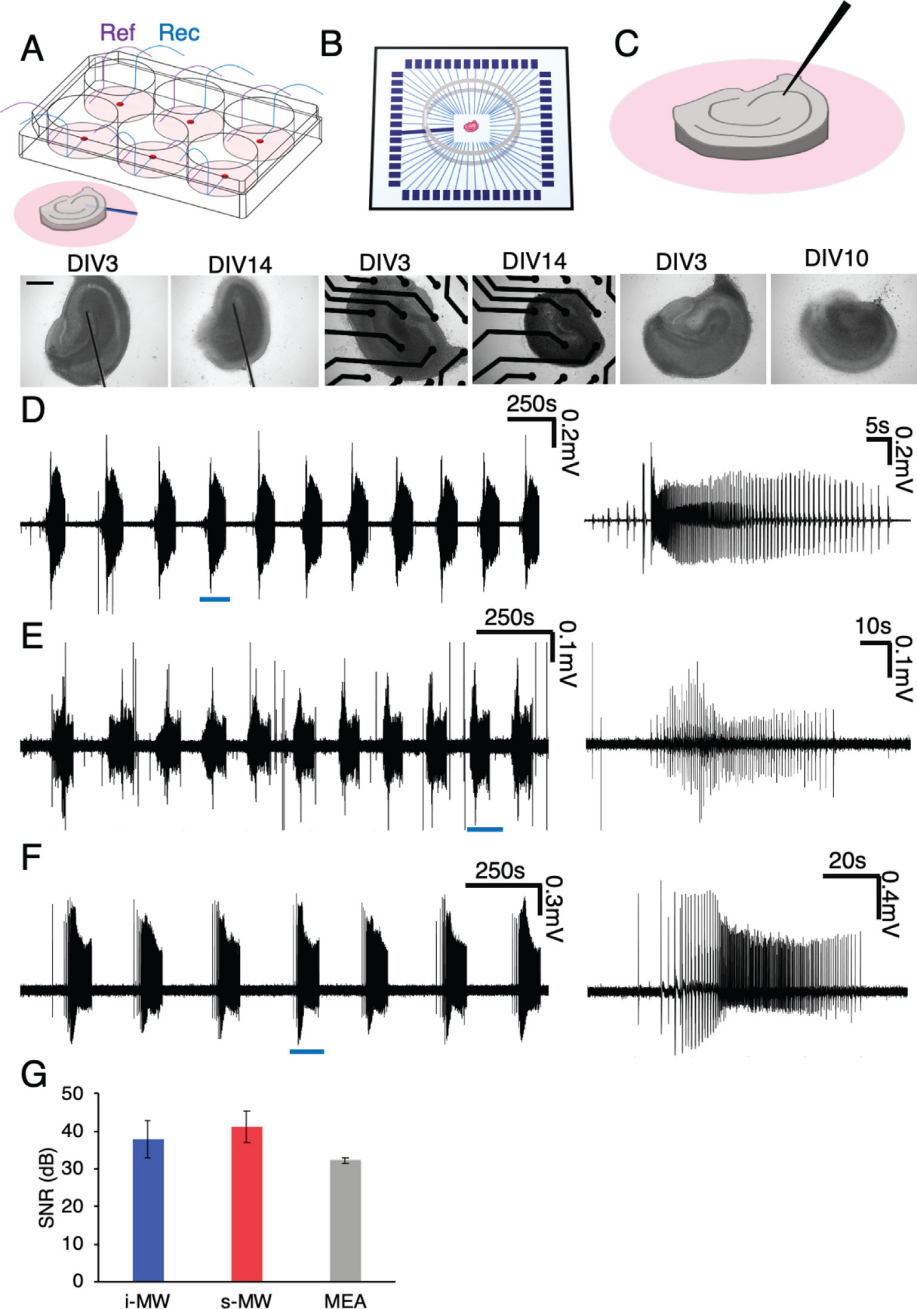
Three local field potential recording platforms. A, B, and C are schematic representations of micro-wire, MEA, and single electrode recording systems respectively. Bright-field images of slices at different days in-vitro are provided at the bottom of each schematic representation of the recording system (scale bar, 500μm). D, E, and F are sample LFP recordings obtained from OHCs cultured on micro-wire plate, from culture recorded by single electrode insertion, and from a slice cultured on a MEA device respectively. Traces on the right are the zoomed in window representing one ictal event. G. Signal-to-noise ratio of signals collected from three recording modalities are shown and no significant difference is detected.

We collected LFP recordings with three different recording modalities described in the methods section which are schematically represented in Figure 1A–C. In order to record from s-MWs, culture plates were transferred to the recording chamber, and to record from cultures on coverslips through the i-MW, the coverslip was transferred to a recording interface. Sample electrical recordings are provided in Figure 1D–F from s-MW, i-MW, and MEA device, respectively. Recordings in which seizures were detectable were chosen for analysis. Seizures are defined as bursts that lasted at least 10 s with amplitudes significantly higher than noise and spike frequency of greater than 2 Hz. The signal-to-noise ratios of s-MW, i-MW, and MEA recordings were all >30 dB (Figure 1 G, n = 3 recordings, differences were not statistically significant). In all three recording modalities (s-MW, i-MW, and MEA) large-area, low-impedance reference electrodes were used (in s-MW and MEA, large area reference electrodes were made from same material as recording microelectrodes, while for i-MW, Ag/AgCl reference was used). In all recording modalities, reference electrodes were located sufficiently far from the slice that the epileptiform activity at reference point was not detectable at reference point. We therefore do not expect that reference electrodes influenced our results.

### 3.2 Seizure stability and jitter

In order to investigate the time dependence of seizure variability, we introduced jitter analysis. Jitter is defined as the average difference in spike timings between any two seizures.

**Figure 2:**
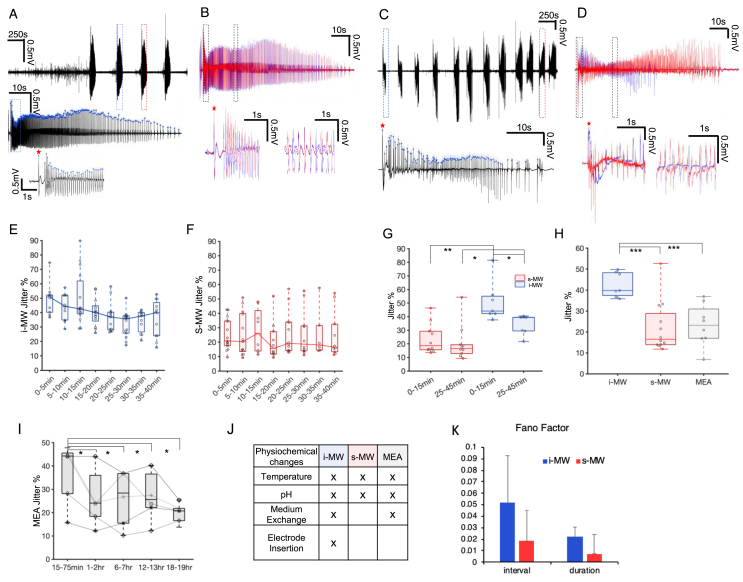
Average jitter percentage calculation and comparison among three recording systems. A, C Examples of recording from s-MW and i-MW respectively are presented in black trace. Zoomed-in window of a single ictal event which is indicated by blue dashed box is provided at the bottom for both examples. B, D Two ictal events that are specified by blue and red dashed boxes in A and C respectively are overlaid according to the alignment spike (red and blue trace). Two zoomed-in time windows at different time points of the recording are presented at the bottom of these two traces for both s-MW and i-MW (indicated by dashed boxes). Blue arrows indicate the spikes and red star specifies the alignment time point. Jitter between two selected events in B and D are 11% (low jitter) and 61% (high jitter), respectively. E, F Jitter evolutions within 1 h time window of recordings from i-MW and s-MW are illustrated in box plots. Values of jitter are binned into 5 min intervals. The whiskers represent the range of the data. The 25^th^ and 75^th^ percentiles of the samples are contained within the top and bottom of the boxes. The line in the middle represent the median. G Jitter at the first 15 min and the last 15–20 min of the recordings are averaged and compared through paired t-test both for s-MW and i-MW samples. The initial and last 15–20 min then compared between two recording modalities through student t-test (p-value ∗ = 0.01, p-value ∗∗ = 0.001, n = 7 recordings, 7 slices for i-MW, and n = 10 recordings, 5 slices for s-MW). H Average jitter percentages were compared among three devices during the more stabilized period and after first 15 min of the recording. The highest value was obtained from i-MW (Student t-test, p-value ∗ <0.04, p-value ∗∗ = 0.001, n = 8 recordings, 5 slices from MEA device, n = 7 recordings, 7 slices from i-MW setting, and n = 12 recordings, 5 slices from s-MW setup). I Average percentage of jitters are calculated within a 1 h time window after 15 min, 1, 6, 12, and 18 h of media exchange. Student t-test was applied to the jitter values of different time points post media exchange. Average jitter percentage is significantly higher during the 1 h time window after 15 min post media changing compared to the other time points (p-value ∗ <0.05, n = 5 slices). J The table summarizes the external disturbances that are present for all three recording platforms. K Duration and interval of seizure-like activities measured from the recordings and normalized fano factors (ratio of variance over mean) are compared between i-MW and s-MW platforms. Fano factors of seizure durations are significantly higher from i-MW recordings (student t-test, p-value < 0.05).

Seizures often began with a large amplitude spike followed by a high frequency of spiking which eventually slows down and becomes less frequent until the seizure terminates. Alternatively, some seizures began with a gradual increase in the population spike rate, reaching a high frequency, and similarly gradually fading away towards the end of the seizure. Similarity in the seizure shapes was quantified by detecting the spikes within all the seizures during one recording. Once the spiking times were measured, we were able to compare the seizure stability during 30–60 min of recording by aligning the seizures to one another. For each of the ictal events, one spike was considered as the alignment spike. For instance, for those seizures starting with a large amplitude spike, this large peak time was considered as the alignment time point. Spike intervals were calculated and the jitter was measured according to Eq. (1). Figure 2. A-D demonstrates our method for calculating jitter percentage and shows examples of low and high jitter between pairs of seizures (Figure 2B, D, respectively).

For recordings from the s-MW, 6-well plates were transferred to a mini-incubator. This transfer results in relatively slow transient alterations in pH balance and temperature. In the i-MW experiment, the pH and temperature transients are faster due to the interface chamber and perfusion system. However, in this recording approach the culture was transferred from the culture medium to the ACSF, and electrode was inserted into the tissue at the time of experiment. To illustrate the effect of these changes, a moving window was used to calculate dependence of recording time on jitter. The duration of the window and the moving step size were assigned least 2 ictal events and the moving step size was determined such that at each step one event was switched. Similar analysis was done on recordings obtained from i-MW. Only the recordings that had at least one ictal event in their first 15 min were considered in this analysis. The evolution of jitter was then demonstrated by binning the data into 5 min intervals (Figure 2E, F). The jitter values in the first 15 min of the recording and the last 15–20 min of the recording were compared and it was significantly higher for i-MW during the initial 15 min of the recording (n = 7 recordings, 7 slices for i-MW, paired t-test p = 0.04, and n = 10 recordings, 5 slices for s-MW, paired t-test p = 0.09, Figure 2 G). The initial and final 15–20 min of the recordings from i-MW and s-MW were then compared between the two recording modalities through student t-test. The jitter showed significantly higher values for i-MW recordings.

To compare the stability and consistency of ictal events on three recording platforms, jitters were measured and compared among all the three recording modalities. Time windows immediately after media exchanges on MEAs and within the first 15 min of i-MW and s-MW recordings were excluded from this comparison. The low value of jitter demonstrates that once the organotypic hippocampal cultures are unperturbed and the environmental conditions are stabilized, seizures have similar waveforms. Interestingly, when jitter from all recording approaches were compared together, i-MW recording setup represented the highest jitter as compared to the s-MWs and MEA devices (Figure 2 H).

The MEA recording platform allowed us to perform long-term recordings of activity of OHCs. To evaluate the effect of media changes, we analyzed the recordings obtained from OHCs at different time steps post media exchange. A 1 h time window was considered beginning at 15 min, 1, 6, 12, and 18 h after media replacement. The jitter was compared at these time points and the jitter was significantly higher during the 1 h time window after 15 min post media exchange. The jitter dropped and seizures stabilized after 1 h in the incubator, but the jitter did not change significantly between 1 and 18 h in the incubator (Figure 2 I).

In Figure 2J, we summarized the external perturbation caused by 3 recording modalities: 1. The change in pH and temperature which were caused by transferring of plate to mini-incubator, 2. Medium exchange, and 3. Electrode insertion. To further address the issue of electrode insertion, we evaluated the i-MW recording platform by analyzing calcium signals in the area immediately surrounding the inserted electrode.

### 3.3 Damage introduced to the tissue upon electrode insertion

**Figure 3:**
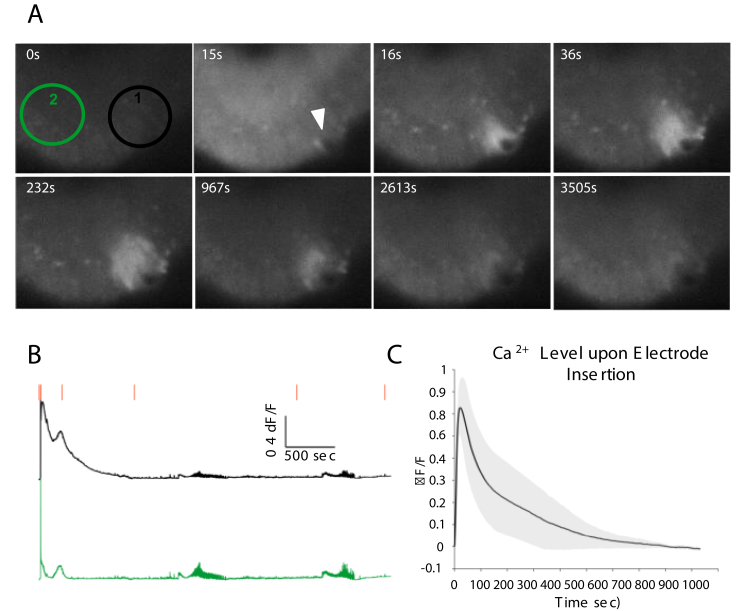
Intracellular [Ca^2+^] elevation upon electrode insertion. A. Frames from an optical recording of the jRGECO1a activity are represented. At 0 s, culture had no activity and calcium signal was at baseline. At 15 s, electrode was inserted into the culture. The insertion spot is indicated by a white triangle. After 1 s, elevated calcium signal is localized to the electrode insertion spot. This elevation in calcium signal reaches the maximum coverage around the site of electrode insertion after about 30 s and gradually decreases. By approximately 900 s, the signal is back to baseline. B. Two ΔF/F traces illustrated in green and black colors obtained from the ROIs specified in A. 0 sec frame, are provided respectively. Red bars are corresponding time points of the presented frames in A. C. ΔF/F signals obtained from ROI1 are subtracted from ROI2, aligned by the time of electrode insertion and averaged. Standard deviation is depicted as gray shadow around the black curve.

We analyzed the damage caused by electrode insertion into OHCs by quantifying the changes in the local calcium level. We used jRGECO1a which is a genetically encoded calcium indicator to optically record the calcium transients as a result of electrode insertion. jRGECO1a was expressed in neurons via syn promoter. Three cultures were recorded optically with a 10X objective for about an hour starting from a few seconds before electrode insertion. Two ROIs were selected from each recording and ΔF/F signal in each was assessed as represented in Figure 3. B. In order to eliminate the spontaneous activity from analysis, signal acquired from unaffected area was subtracted from the signal obtained from the region under insertion. Finally, signals from three devices were aligned based on the time of the electrode insertion and the average calcium signal vs time was calculated and shown in Figure 3 C. Upon electrode insertion a local increase in the intracellular calcium occurred that progressively increased in the area reaching the maximum extent after about 20 s. This elevated calcium then decayed back to within 2% of baseline after approximately 15 min.

### 3.4 Jitter evolution in MEA recordings reveals spontaneous state transitions

**Figure 4:**
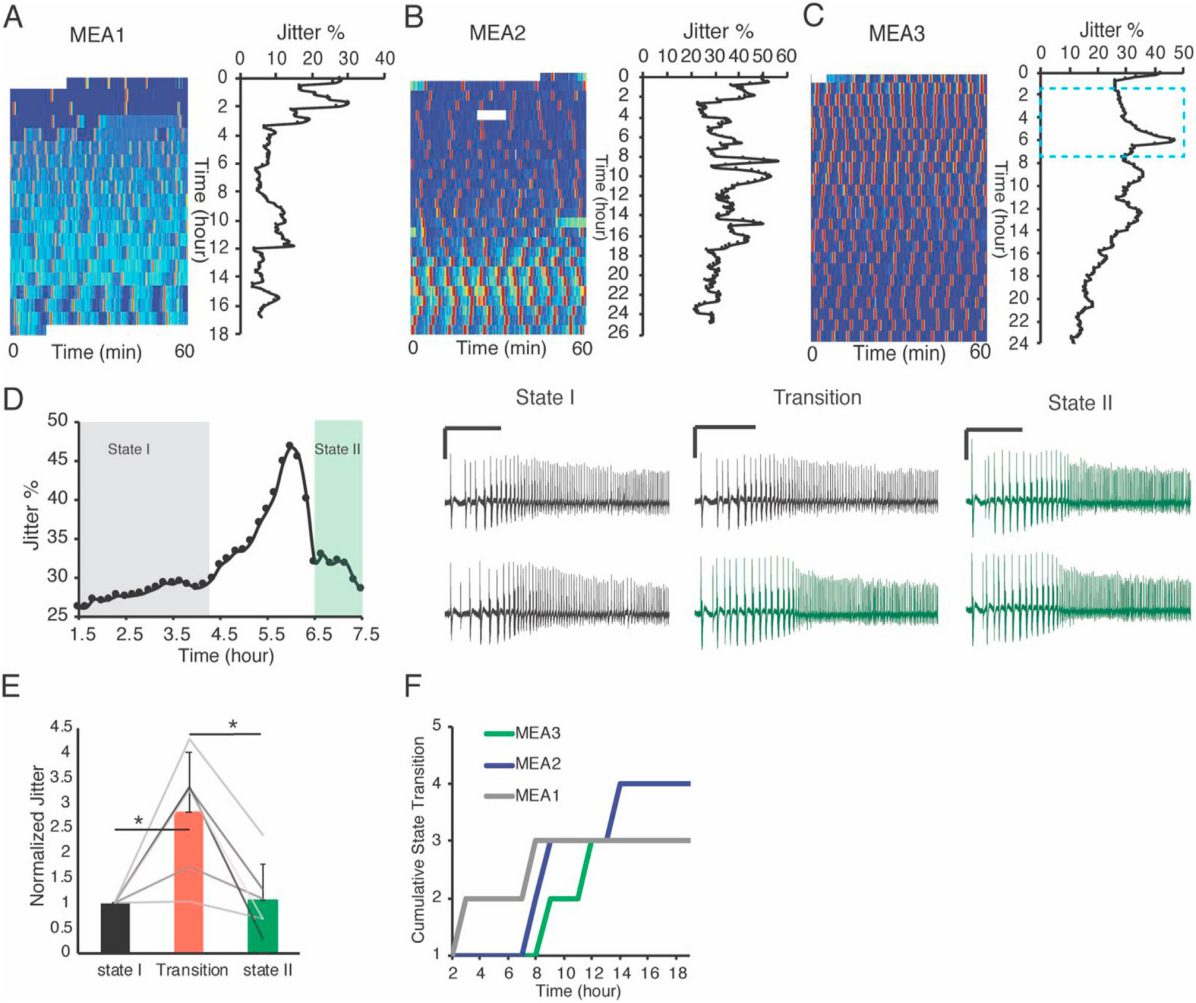
Natural state transitions in OHCs. A, B and C Color coded raster plots of the activity of three OHCs cultured on MEA devices during 17–25 h of chronical LFP recording are provided. The graphs on the immediate right side of these raster plots, represent the jitter evolution measured using a sliding 1 h window. The window is shifted every 10 min and average jitter percentage is calculated at each step. D. Enlarged view of region enclosed by the blue dashed rectangle in C, shows a state transition. State I and state II are depicted by highlighted boxes in gray and green respectively. Two example events are selected from each state and are shown on the right side (scale bars, 0.5 mV and 10 s). E. Two seizures were chosen from the state before transition and their jitter was calculated (state I jitter). Similarly, two seizures were chosen from the state after the transition period, and their jitter was also calculated (state II jitter). Jitter between a seizure in the state I and a seizure in state II was calculated as well, and classified as Transition jitter (n = 6 transitions within different MEAs, ∗p < 0.05, student t-test). F Cumulative plots of the state transitions within three MEA devices are depicted.

The number of ictal events, the intervals between seizures and the seizure shapes undergo time-dependent evolution in each culture. To better illustrate this spontaneous behavior, we created color coded raster plots from chronic recordings of 17–25 h duration for three MEA devices (Figure 4A–C). In the raster plots, dark blue represents no activity and dark red represents high frequency population spiking lasting for at least 10 s. A 1 h window was shifted with step size of 10 min over recordings and at each step the jitter was calculated. This continuous analysis of changes in the jitter allowed us to better track the jitter evolution (plots in Figure 4A–C). The time zero is the time of media replacement. In our previous analysis of MEA recordings, we showed that media exchange increases instability in the seizure behavior for about an hour. After this first hour, we found there were multiple time points that cultures experienced transient instability in the seizure jitter. However, these increases in the jitter values were temporary and either gradually or immediately decreased to their “stable” mode even though the frequency and pattern of spiking were different from the previous stable state (Figure 4D). Jitter values were significantly lower during stable states (with constant seizure waveform) compared to state transitions (periods when seizure waveform underwent change) (Figure 4E). To quantify how many times a culture went through spontaneous state transitions without external perturbations, the standard deviation of jitter percentages throughout each recording was measured and a state transition was considered to have occurred when threshold of 2 or more standard deviations was exceeded (Figure 4F). To investigate the correlation between jitter values and the interval and duration of ictal events, standard deviation of seizure durations, and intervals between events were calculated for one of the MEA devices. The Pearson's linear correlation coefficient was 0.32 between jitter and standard deviation of duration of ictal events, and the p-value was 0.0003. The correlation coefficient for jitter and standard deviation of interval between ictal events was 0.08 and p-value was 0.36. This signifies that jitter analysis is a better method to identify state transitions than analysis of seizure durations or intervals.

## 4 Discussion

In this study we explored whether the electrical recording method affects variability of data obtained from an *in vitro* epilepsy model. We have shown that seizure stability can be affected by an external perturbation. We classified the potential external perturbations (causes of increased variance) into changes in pH, temperature, composition of recording medium, and insertion of an electrode. Figure 2J summarizes perturbations that slice cultures experienced when recorded via i-MW, s-MW, and MEA methods. Inserted electrode method, while the most widely used of the three, is also the one that causes the most changes to neuron microenvironment. In i-MW, slices are placed into a simplified recording medium, and thus a change in the chemical environment as well as temperature and pH transients during transfer to the recording chamber. Jitter of i-MW recorded seizures drops significantly after the first 15 min but remains higher than jitter in s-MW recorded cultures even after one hour of recording. We can therefore conclude that there are at least two sources of variability affecting i-MW recordings, with different time constants (one on the order of minutes, the other on the order of hours). Temperature and pH transients also affect s-MW method of recording due to transfer of culture plates from the incubator to the recording chamber. However, jitter for this method starts at a low value even in the first 15 min of recording. This result suggests that temperature and pH transients are not an important source of variability for seizures in organotypic hippocampal cultures.

MEA recorded slice cultures, on the other hand, have highly variable seizures for the first 75 min of recording, and then jitter drops to a similar level as in the s-MW method. We can therefore conclude that the medium exchange that occurred just before initiation of the 24 h MEA recordings is the major source of this jitter. Considering that elevated Ca^2+^ lasts approximately 15 min after electrode insertion, damage caused by the electrode is likely the cause of the variability/high jitter in the first 15 min of i-MW recordings. Prolonged elevation of intracellular calcium can causes neuronal toxicity. Consequently, in addition to the acute damage that the electrode insertion can cause, the prolonged calcium elevation can also result in changes to the intra- and extracellular environments (Potter et al., 2012; Kozai et al., 2015; Ravikumar et al., 2014; McConnell et al., 2009; Wellman and Kozai, 2017; Biran et al., 2005; Eles et al., 2018). But, comparison of i-MW and MEA method suggests that it is actually the placement of the slice into unfamiliar recording medium that causes most of longer-lasting variability. In i-MW method, the recording medium is ACSF which lacks many of the amino acids, vitamins, and proteins present in culture medium in which the slices were maintained (Liu et al., 2017). Therefore, it may not be surprising that transition to ACSF may have a strong effect on spontaneous seizures. On the other hand, variability caused by refreshing the culture medium prior to MEA recordings is more surprising. Cells within the slice consume nutrients in the culture medium and release biomolecules, including trophic factors, into it. Culture medium that has been ‘conditioned’ by cells such as astrocytes has long been recognized as superior to fresh medium in terms of supporting neuronal survival (Banker et al., 1998). Our data suggests that the medium conditioning by the hippocampal slices is also an important factor in the stability of spontaneously occurring seizures in this *in vitro* model.

We have shown that the effect of medium change on the recorded signal is quite significant and long term and can cause substantial variability in seizure waveforms. This is particularly important for perfusion systems where the culture medium is constantly replaced with fresh medium. The effect of electrode insertion on the consistency of seizure generation, on the other hand, was shown to be rather short and could be avoided by simply allowing slices to stabilize for 15 min or more. These results are surprising, as one would expect that the electrode insertion would be the major source of instability, while placing slices into fresh medium could have been expected to actually increase stability, rather than cause a long term decrease as shown by our results. Understanding the experimental conditions that may improve stability of spontaneous epileptiform activity may be critical in robust applications of this model of epilepsy.

Since the stability of the chemical microenvironment (medium composition) is critical, it may be difficult to achieve low variability with i-MW method. This method requires the use of an open recording chamber to place the microelectrode into the slice. This in turn necessitates perfusion to maintain constant osmolarity, pH, and temperature of the recording medium. Perfusion requires a large volume of the recording solution, making it impossible to use medium that was conditioned by the slice cultures. Thus, if low seizure variability is desired in an experiment, s-MW or MEA methods should be used.

We also carried out long-term recordings with MEAs and evaluated jitter evolution for up to 20 h. These recordings revealed 3–4 transient periods of high jitter occurring at random times during the 20 h. We examined seizure waveforms before and after these high jitter periods. We found that seizure waveform changed during the high jitter period, and settled into a new, stable pattern during the low jitter period that followed. Hence, we hypothesized that high jitter periods represent spontaneous transitions of the state of neural circuit in the slice cultures.

Seizure waveform depends on the mechanisms by which it is generated, propagated, sustained, and terminated. A transient increase in jitter, which signifies transition from one stable seizure waveform to another, implies that a change has occurred in one of these mechanisms. It has been shown via chronic intracranial EEG recordings that seizure similarity varies over time and with circadian rhythm, and that seizures which occur closer in time to one another tend to be more similar (Schroeder et al., 2020). The analysis technique used by Schroeder et al., termed “dynamic time warping”, requires spatial and frequency information available from multi-channel recordings to analyze similarity between seizures. Jitter analysis, on the other hand, is capable of comparing seizures, and finding state transitions, using single electrode information. In this study, we showed that seizure state transitions occur in the reduced slice preparation in vitro, suggesting a role for local mechanisms. State transitions may be caused by progression of epileptogenesis, which, in organotypic hippocampal cultures, may include axon sprouting and reorganization of neuronal circuitry. These changes in the network may result in generation of seizures with different patterns. There may also be ongoing cell death in slices due to seizures (Berdichevsky and Dzhala, 2012), which in turn may cause compensatory changes in the network.

Machine learning has been used to estimate the phase of oscillations in the brain (McIntosh and Sajda 2020). Timing of spikes within a seizure may be thought of as phase information that may also be analyzed via machine learning. However, quantification of similarity between spontaneous seizures may not lend itself to machine learning analysis. This is due to lack of a priori knowledge about timing of steady states and transitions in a given recording, which may make it difficult to train the phase recognition algorithm. Another disadvantage of phase correlation analyses (Netoff and Schiff 2002) for the purpose of seizure similarity quantification is that a time delay of any single seizure phase-containing feature (such as a spike) will affect phase delays detected from the following spikes and result in an incorrectly low assessment of similarity. In our analysis, we calculated jitter between consequent pairs of spikes from beginning of seizure to the end, so that a time shift of one spike does not affect analysis of subsequent spikes. This is also a feature of the significantly more complex dynamic time warping technique (Schroeder et al., 2020). Jitter analysis is a relatively simple, quick, and robust technique to identify and quantify state transitions via assessment of similarity between seizures, and may be adopted for future studies seeking to understand state transition mechanisms in epileptic networks.

## 5 Conclusions

The choice of *in vitro* electrical recording method plays a large role in the variability of seizure duration, interval, and waveform. Most important sources of variability include electrode insertion and change of the recording medium. Increased variability due to electrode insertion has a relatively short time constant on the order of minutes, and its influence on recording can be minimized by allowing recorded slices to stabilize for approximately 15 min. On the other hand, change of medium induces larger variability lasting longer than an hour. Recording of slices in conditioned medium with substrate-integrated electrodes was found to produce the lowest variability and require no stabilization time. Hippocampal slice cultures also underwent state transitions that occurred spontaneously, and that were characterized by transient increases in variability. The presence of these spontaneous transients indicates that at least some of the variability is independent of the electrical recording method.

## Declarations

### Author contribution statement

Ghiasvand S.: Conceived and designed the experiments; Performed the experiments; Analyzed and interpreted the data; Wrote the paper.

Dussourd C.: Analyzed and interpreted the data; Contributed reagents, materials, analysis tools or data.

Liu J., Song Y.: Performed the experiments; Contributed reagents, materials, analysis tools or data.

Berdichevsky Y.: Conceived and designed the experiments; Analyzed and interpreted the data; Wrote the paper.

### Funding statement

This work was supported by the 10.13039/100000065National Institute of Neurological Disorders and Stroke of the 10.13039/501100003653National Institute of Health (R21/R33NS088358).

### Data availability statement

Data associated with this study has been deposited at Github under the URL: https://github.com/Chris-Dussourd/Epilepsy-Jitter-Code.

### Declaration of interests statement

The authors declare no conflict of interest.

### Additional information

No additional information is available for this paper.
